# Investigating the effect of proteoglycan 4 on hyaluronan solution properties using confocal fluorescence recovery after photobleaching

**DOI:** 10.1186/s12891-019-2469-4

**Published:** 2019-02-26

**Authors:** Adam K. Bloom, Michael L. Samsom, Suresh C. Regmi, Bridgett L. Steele, Tannin A. Schmidt

**Affiliations:** 1 Biomedical Engineering Graduate Program, University of Calgary, Calgary, AB Canada; 2 Faculty of Kinesiology, KNB 426, 2500 University Dr. NW, University of Calgary, Calgary, AB T2N 1N4 Canada; 3 Biomedical Engineering Department, University of Connecticut Health Center, Farmington, CT USA

**Keywords:** Hyaluronan, Proteoglycan 4, Confocal FRAP, Tracer diffusion, Solution network

## Abstract

**Background:**

The objective of this study was to use confocal fluorescence recovery after photobleaching (FRAP) to examine the specific and dose-dependent effect of proteoglycan 4 (PRG4) on hyaluronan (HA) solutions of different molecular weight; and assess the effect of reduction and alkylation (R/A) of PRG4 on its effects on HA solutions.

**Methods:**

Confocal FRAP was used to determine the diffusion coefficient of fluorescein isothiocyanate (FITC)-dextran tracer (D_t_) through 1500 kDa and 500 kDa HA solutions (0–3.3 mg/ml) ± PRG4 or a control protein, bovine serum albumin (BSA), at physiological (450 μg/ml) or pathophysiological (45 μg/ml) concentrations. The effect of PRG4 or R/A PRG4 on 1500 kDa HA solutions was also investigated. Empirical constants obtained from fitting data to the universal scaling equation were used to calculate the average distribution of apparent mesh sizes.

**Results:**

PRG4 at both 45 and 450 μg/ml slowed the diffusion of the FITC-dextran tracer for all concentrations of HA and caused a decrease in the apparent mesh size within the HA solution. This effect was specific to PRG4, not observed with BSA, but not dependent on its tertiary/quaternary structure as the effect remained after R/A of PRG4.

**Conclusions:**

These results demonstrate that PRG4 can significantly alter the solution properties of HA; PRG4 essentially reduced the permeability of the HA network. This effect may be due to PRG4 entangling HA molecules through binding and/or HA crowding PRG4 molecules into a self-assembled network. Collectively these findings contribute to the understanding of PRG4 and HA interaction(s) in solution and therefore the function of SF in diarthroidal joints.

## Background

Hyaluronan (HA) is a vital macromolecular component of synovial fluid (SF) with several important functions. HA is a negatively charged biopolymer composed of alternating D-glucuronic acid and N-acetylglucosamine that forms dynamic networks in solution [1]. HA exists in SF at molecular weights (MW) between 0.2 to 6 MDa, and concentrations of 1–4 mg/ml [2, 3]. A major role of HA in SF is to impart fluid viscosity and elasticity to help transfer loads across the cartilage within the articulating joint. HA has also been shown to effectively reduce friction in dose-dependent manner at a cartilage-cartilage biointerface under boundary mode lubrication [4, 5].

Proteoglycan 4 (PRG4) is a mucin like glycoprotein, with extensive O-linked glycosylation and an apparent MW of ~ 460 kDa. It is also present in SF and covers the surface of articulating cartilage [6, 7]. PRG4 is a flexible rod ~200 nm in length and 1-2 nm in width, and its hydrodynamic diameter as measured by light scattering has been reported to be ~ 200 nm as well [8, 9]. PRG4 has been reported at an average concentration of 287 +/− 31.8 μg/ml in healthy human SF, though it can vary from 129 to 450 μg/ml [10]. PRG4 effectively reduces friction in a dose-dependent manner at a cartilage-cartilage biointerface under boundary lubrication, [4] as well as at cartilage-glass and latex-glass surfaces [6, 11, 12]. PRG4 is capable of dimerization via intermolecular disulfide bonds and exists in SF in both monomeric and dimeric forms [13]. Reduction and alkylation (R/A) of PRG4, causing disruption of intra and inter molecular disulfide bonds, has been shown to reduce multimers into monomers and release small fragments from the PRG4 structure (~ 70 kDa) [13]. This results in a significant reduction in binding of PRG4 to the surface of articular cartilage and an associated reduction in its cartilage boundary lubrication [7, 12, 14].

PRG4 and HA function synergistically as lubricants at the cartilage surface, and possibly in solution within SF. When combined in solution at physiological concentrations PRG4 and HA reduce friction at a cartilage-cartilage biointerface under boundary lubrication to lower levels than either alone, approaching the lubrication of healthy SF [4, 5, 15]. Additionally PRG4 has been shown to enhance/alter the viscosity of HA solutions [16]. This functional synergism has been demonstrated with various MW HA without any significant variation in lubricating ability [5]. This synergism suggests a functional interaction between HA and PRG4 at the cartilage surface, and possibly in solution as well. However interactions of macromolecules may not be the same at a surface and in solution.

Previous studies have attempted to elucidate the mode of interaction of PRG4 and HA in solution [2, 5]. An electrophoretic mobility shift assay provided evidence of a weak PRG4 + HA interaction [5]. Additionally, a multiple-particle-tracking microrheology technique has been used to study the effect PRG4 has on the biophysical properties of SF (a semi dilute HA solution) and provide evidence for an interaction in solution [2]. Experimentation performed on healthy as well as PRG4-deficient SF suggested that PRG4 creates a network of “entanglements” within HA-containing SF, resulting in an increased relaxation time for SF [2]. However, the specific mechanism and concentration dependence of this interaction has yet to be determined. A more detailed understanding of HA and PRG4 interaction in solution could help explain the molecular basis of the biophysical properties of normal and pathological SF. Accordingly, an experimental technique which allows us to probe the biophysical properties of complex HA and PRG4 solutions would be valuable.

Confocal fluorescence recovery after photobleaching (FRAP) is a microscopic technique that has been used to investigate solution properties and molecular networks of HA. Confocal FRAP provides a powerful tool for studying concentrated and complex polymer solutions in the absence of shear stress [17]. Gribbon et al. [18] used FRAP to determine how electrolyte concentration and pH effect the hydrogen and electrostatic intramolecular bonds within the repeat sugar subunits of HA. The resulting change in intramolecular bonding was shown to change the stiffness, and contraction of the HA molecules, and thus network formation. Due to confocal FRAP’s ability to reveal details about the structure HA networks it is an ideal method to evaluate the specific and dose-dependent effect PRG4 has on HA solution properties.

The objectives of this study were to 1) use confocal FRAP to examine the specific and dose-dependent effect of PRG4 on HA solution networks by analyzing the diffusion of a fluorescein isothiocyanate (FITC)-dextran tracer through HA solutions of different MW, and 2) assess the effect of an altered tertiary/quaternary PRG4 structure, through R/A, on the observed effects on HA solutions at different concentrations.

## Methods

### Materials

**Fig. 1:**
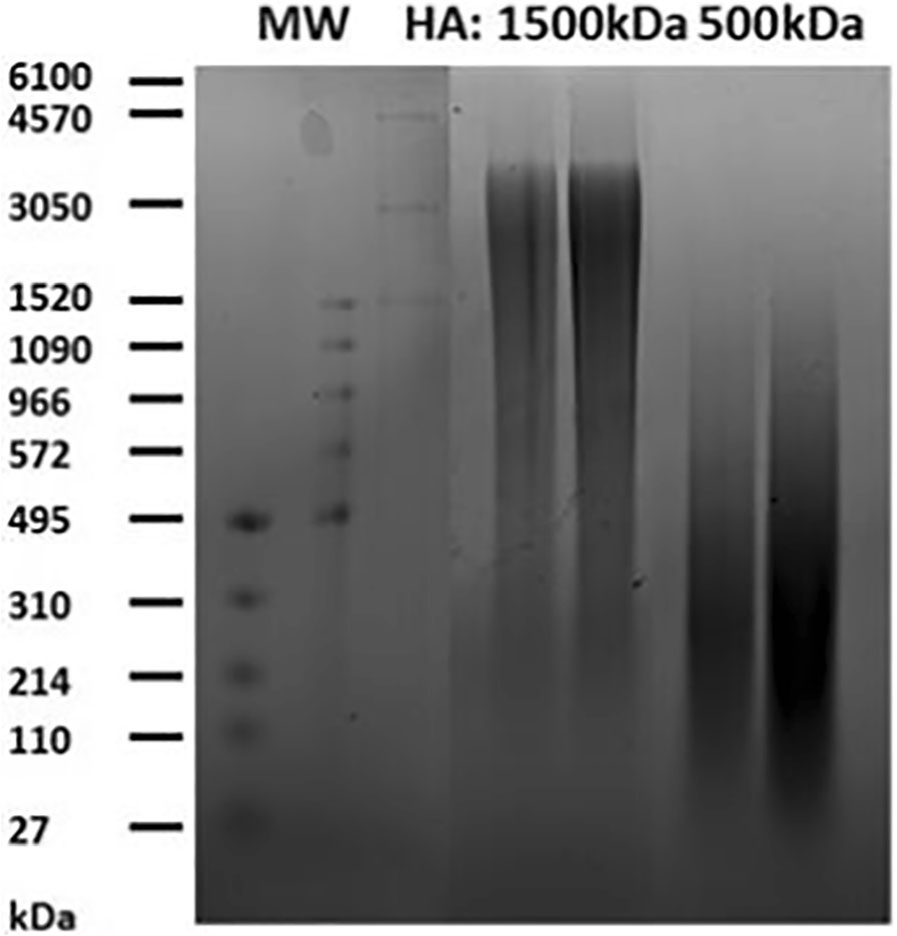
MW characterization of HA used in confocal-FRAP studies via agarose gel electrophoresis

HA, 1500 kDa and 500 kDa, was obtained from LifeCore Biomedical (Chaska, MN, USA), and the MW was qualitatively confirmed by 1% agarose gel electrophoresis (Fig. 1) [5]. FITC-dextran (MW = 2000 kDa) and bovine serum albumin (BSA) were obtained from Sigma-Aldrich (St. Louis, MO, USA). The hydrodynamic radius of the FITC-dextran tracer was determined using dynamic light scattering to be 19.50 ± 1.29 nm (mean ± SEM, *n* = 3), which is in good accordance with reported values [18]. All samples were prepared in calcium and magnesium free phosphate buffered saline (PBS).

PRG4 was prepared using previously described methods [4]. Briefly, articular cartilage disks were harvested from bovine stifle joints obtained from a local abattoir (Calgary, AB, Canada) and cultured in the presence of transforming growth factor-β1 [4]. The PRG4 was then purified from conditioned media using anion exchange chromatography and centrifugal filtration [4]. The purity was confirmed using 3–8% Tris-Acetate SDS-PAGE followed by protein stain and western blotting. The concentration was then determined by bicinchoninic acid assay (Thermo Fisher; Rockford, IL, USA). R/A PRG4 was prepared by incubating in PBS with 10 mM dithiothreitol for 2 h at 60C and pH = 8.5 and then 40 mM iodoacetate for 2 h at room temperature [13]. The R/A PRG4 was then buffer exchanged into PBS through dialysis overnight with frequent changes. R/A of PRG4 was confirmed through SDS-PAGE followed by protein staining (data not shown).

### Sample preparation

All samples below were prepared at room temperature, fresh on the day of use and not stored afterwards. HA was weighed out then reconstituted in PBS to form the HA solutions to which the FITC-dextran was added, then PRG4/BSA was added (as described below) if appropriate. Samples were then vortexed briefly then allowed to nutate for 2 h at room temperature (stored with tin foil to protect from light) prior to Confocal FRAP measurements.

#### HA concentration series

1500 kDa and 500 kDa HA solutions were prepared in experimental sets of concentrations 0, 0.1, 0.3, 1 and 3.3 mg/ml. FITC-dextran was added into every HA solution at a final concentration of 0.1 mg/ml.

#### HA ± PRG4

Each prepared HA solution above was divided into two samples. Half of the HA samples were added to a dried mass of PRG4 to a final concentration of either 450 or 45 μg/ml to make the HA + PRG4 solution series, while the other half of the HA solutions remained unaltered.

#### HA ± BSA

HA ± BSA solutions were made in same way as HA ± PRG4 solutions except powdered BSA was added instead of PRG4.

#### HA + PRG4 vs. HA + R/A PRG4

Each prepared HA solution was divided into two samples. Each was added to a dried mass of PRG4 or R/A PRG4, to a final concentration of either 450 or 45 μg/ml, to make the HA + PRG4 and HA + R/A PRG4 solution series respectively.

### Confocal FRAP protocol

Samples were mounted and sealed onto concave depression slides (Pearl, China) and subjected to confocal FRAP experiments performed on a Zeiss LSM780 scanning confocal microscope with a 40x objective, essentially as described previously [18]. The microscope was set to a pixel size of 0.19 μm and a pixel dwell time of 0.79 μsec. The pinhole was set to the maximum value. A 96.5 × 96.5 μm area was monitored by a 489 nm 100 mW diode laser at 0.2% maximum power for 20 scan cycles. The 489 nm 100 mW diode laser was then used in tandem with a 405 nm 100 mW diode laser, both at 100% maximum power, to bleach a central circle with a diameter of 19 μm for 20 scan cycle iterations. The post bleach sample was then monitored by the 489 nm 100 mW diode at 0.2% maximum power for a total of 600 cycles.

### Data analysis

The obtained images were analyzed by directly fitting the time development of the bleaching profile to the Bessel expansion solution of the cylindrical diffusion equation (Eq. 1) [19].

1 $$ u\left(r,t\right)=\sum \limits_{1=n}^{\infty }{C}_n{e}^{\frac{{\lambda_n}^2 Dt}{R^2}}J\left(\frac{\lambda_nr}{R}\right) $$

Where u is the scaled intensity of the fluorescence, r is the radius, t is time, C_n_ is defined in Eq. 2, J_n_ is the n order Bessel, D is the lateral diffusion coefficient, R is the total radius of the photo bleached area, and λ_n_ is the n^th^ zero to J_0_.

2 $$ {C}_n=\sum \limits_{1=n}^{\infty}\frac{\int_0^Rr{J}_0\left(\frac{\lambda_nr}{R}\right)f(r) dr}{\frac{R^2}{2}{\left[{J}_0\left({\lambda}_n\right)\right]}^2} $$

Where f(r) is the limit condition.

In summary, the image time series were processed with a 3 × 3 Gaussian filter to remove high frequency noise. The fluorescent intensity at the corners of the images was used to scale the images to photobleaching that occurs during monitoring of the images. The radial average of intensity for each image was determined to generate radial intensity plots for every time point which represented the distribution of fluorescent intensity from the center of the image. D was than determined using the Bessel expansion solution of the cylindrical diffusion equation (Eq. 1) to directly fit the time development of the experimental data using a Levenberg–Marquardt algorithm, with the initial condition set to the initial bleaching profile at t = 0.

For all experimental sets (0.1 to 0.3 mg/ml HA) the calculated diffusion coefficients were fit to the tracer diffusion scaling equation (Eq. 3) using a least squares optimization [20].

3 $$ {D}_t={D_t}^0\exp \left(-\beta {c}^{\gamma}\right) $$

Where D_t_ is the lateral diffusion coefficient of the tracer, D_t_^0^ lateral diffusion coefficient of the tracer in PBS (free diffusion coefficient of the tracer), c is the concentration of the polymer, and β and ν are empirical constants [20]. β typically relates to the inter polymer hydrodynamic interaction between the tracer and the polymer matrix, while deviations of ν from 1 relate to contraction of the polymer at high concentrations [18]. When fitting the data to the universal scaling equation, D_t_^0^ was set as a free parameter. The empirical constants from the scaling equations were used to calculate the average distribution of apparent mesh sizes, ξ, using the correlation length relation (Eq. 4) [21].

4 $$ \xi =\left(\frac{d}{\beta}\right){c}^{-\upnu} $$

Where d is the hydrodynamic diameter of the tracer. All calculations and image processing was performed with Matlab® (MathWorks, USA).

### Statistical methods

A total of 4 independent samples were measured for every data point (*N* = 4), with the exception of the HA + PRG4 vs. HA + R/A PRG4 (450 μg/ml) where a total of 5 independent samples were measured (*N* = 5). The diffusion coefficient calculated for each of the 4 independent samples was the result of 6 averaged measurements at randomly chosen points on the concave microscope slide (*n* = 6). These points were chosen at an adequate distance from previous measurements to ensure the new area was not exposed to residual effects of previous photobleaching. All confocal FRAP measurements for an experimental set (i.e. 0 to 3.3 mg/ml ± PRG4) were performed on the same day to reduce extraneous variables. Thus an HA concentration series was compared to an identical HA series, at the same time of day, with the exception of the additive added to the respective HA concentration set (PRG4, BSA or R/A PRG4). Experiments determining D_t_^0^ for 2000 kDa FITC-dextran were also performed individually and specifically for each experimental set. The effect of HA concentration and added protein (PRG4 or BSA) on the diffusion coefficients was assessed, as main effects, using a two factor ANOVA. Data is presented as mean ± SEM.

## Results

### HA ± PRG4

**Fig. 2:**
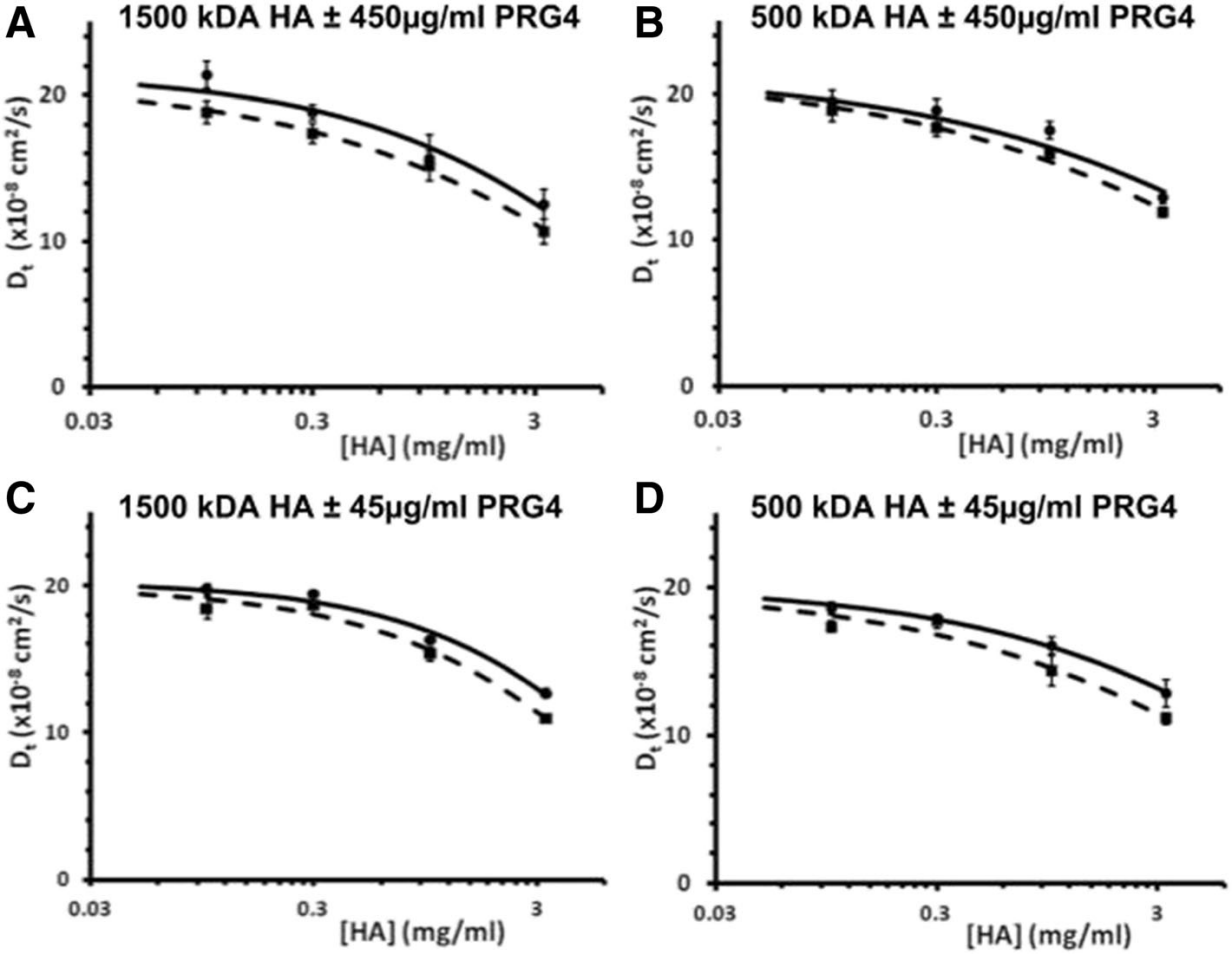
Tracer diffusion coefficients of FITC-dextran (2000 kDa) through 1500 kDa HA solutions ±450 μg/ml PRG4 (▪ for HA+ PRG4, ● for HA) (**a**), 500 kDa HA solutions ±450 μg/ml PRG4 (**b**); 1500 kDa HA solutions ±45 μg/ml PRG4 (**c**), 500 kDa HA solutions ±45 μg/ml PRG4 (**d**). Data points are fit to the universal scaling equation (dashed lines = HA + PRG4, solid lines = HA). For both 1500 and 500 kDa HA (**a**, **b**), the diffusion of the tracer was significantly affected by HA concentration (both *p* < 0.01) and the presence of 450 μg/ml PRG4 (both *p* < 0.05), with no interaction effect (*p* = 0.78 and 0.85 respectively). For both 1500 and 500 kDa HA (**c**, **d**), the diffusion of the tracer was significantly affected by HA concentration (both *p* < 0.01) and the presence 45 μg/ml PRG4 (*p* < 0.01 and *p* < 0.05, respectively), with no interaction effect detected (*p* = 0.18 and 0.646 respectively)

PRG4 at 450 μg/ml slowed the diffusion of the FITC-dextran tracer for all concentrations of HA and therefore had an effect on the HA solution network. For both 1500 and 500 kDa HA (Fig. 2a, b), the diffusion of the tracer was significantly affected by HA concentration (both *p* < 0.01) and the presence of PRG4 (both *p* < 0.05), with no interaction effect (*p* = 0.78 and 0.85 respectively). For all experimental sets there was a clear negative exponential decrease in tracer diffusivity as HA concentration increased. The addition of PRG4 at 450 μg/ml caused an average decrease in D_t_ of 1.592 × 10^−8^*cm*^2^ *s*^−1^, in 1500 kDa HA, and 1.068 × 10^−8^*cm*^2^ *s*^−1^, in 500 kDa HA, both of which were significant (*p* < 0.05). The measured D_t_^0^ for the FITC-dextran tracer was 21.04 ± 0.99 × 10^−8^*cm*^2^ *s*^−1^, and 21.64 ± 1.09 × 10^−8^*cm*^2^ *s*^−1^, for 1500 kDa and 500 kDa respectfully. PRG4 at 45 μg/ml also had an effect on the HA solution network. For both 1500 and 500 kDa HA (Fig. 2c, d), the diffusion of the tracer was significantly affected by HA concentration (both *p* < 0.01) and the presence of PRG4 (*p* < 0.01 and *p* < 0.05, respectively), with no interaction effect detected (*p* = 0.18 and 0.646 respectively). Also similar to above, there was a clear negative exponential decrease in diffusivity for the tracer as HA concentration increased. The addition of PRG4 at 45 μg/ml in caused an average decrease in D_t_ of 1.011 × 10^−8^*cm*^2^ *s*^−1^, in 1500 kDa HA, and 1.263 × 10^−8^*cm*^2^ *s*^−1^, in 500 kDa HA, both of which were significant (*p* < 0.01, 0.05 respectively). The measured D_t_^0^ for the FITC-dextran tracer was 20.04 ± 0.40 × 10^−8^*cm*^2^ *s*^−1^, and 20.18 ± 0.44 × 10^−8^*cm*^2^ *s*^−1^, for 1500 and 500 kDa respectively.

**Fig. 3:**
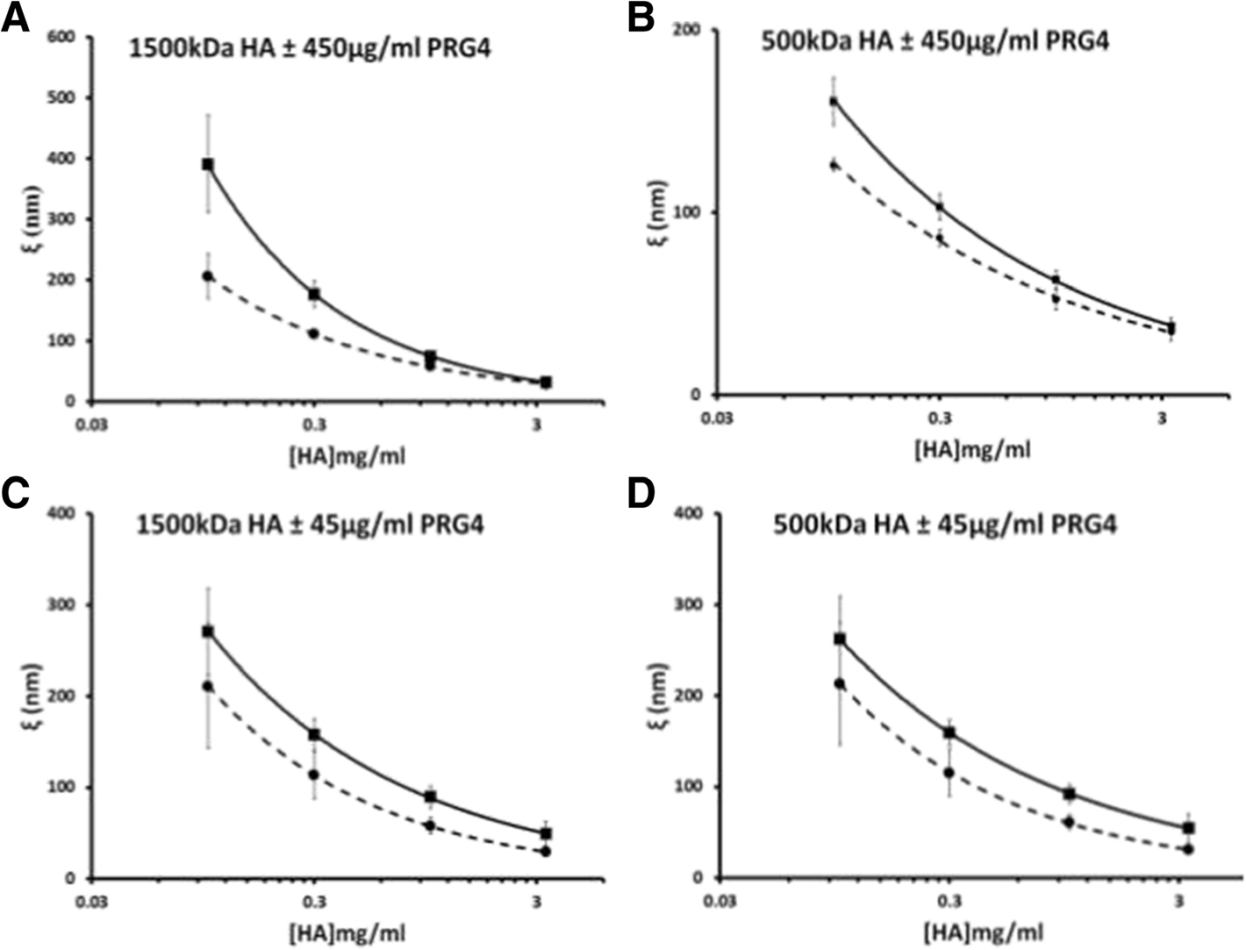
Apparent mesh size distribution (ξ) of 1500 kDa HA ± 450 μg/mL PRG4 (▪ for HA+ PRG4, ● for HA) (**a**), 500 kDa HA ± 450 μg/mL PRG4 (**b**); 1500 kDa HA ± 45 μg/mL PRG4 (**c**), 500 kDa HA ± 45 μg/mL PRG4 (**d**). The empirical constants from the scaling equations were used to calculate the average distribution of apparent mesh sizes, ξ, using the correlation length relation (dashed lines = HA + PRG4, solid lines = HA)

The addition of PRG4 to the HA solutions caused a decrease in the calculated apparent mesh size, or average pore size, within the HA solution network (Fig. 3). As the concentration of HA in solution is increased, the distance separating each molecule decreased according to a negative exponential trend. The variation between HA solutions, with and without PRG4, decreases as the solution becomes saturated with HA at approximately 3.3 mg/ml.

### HA ± BSA

**Fig. 4:**
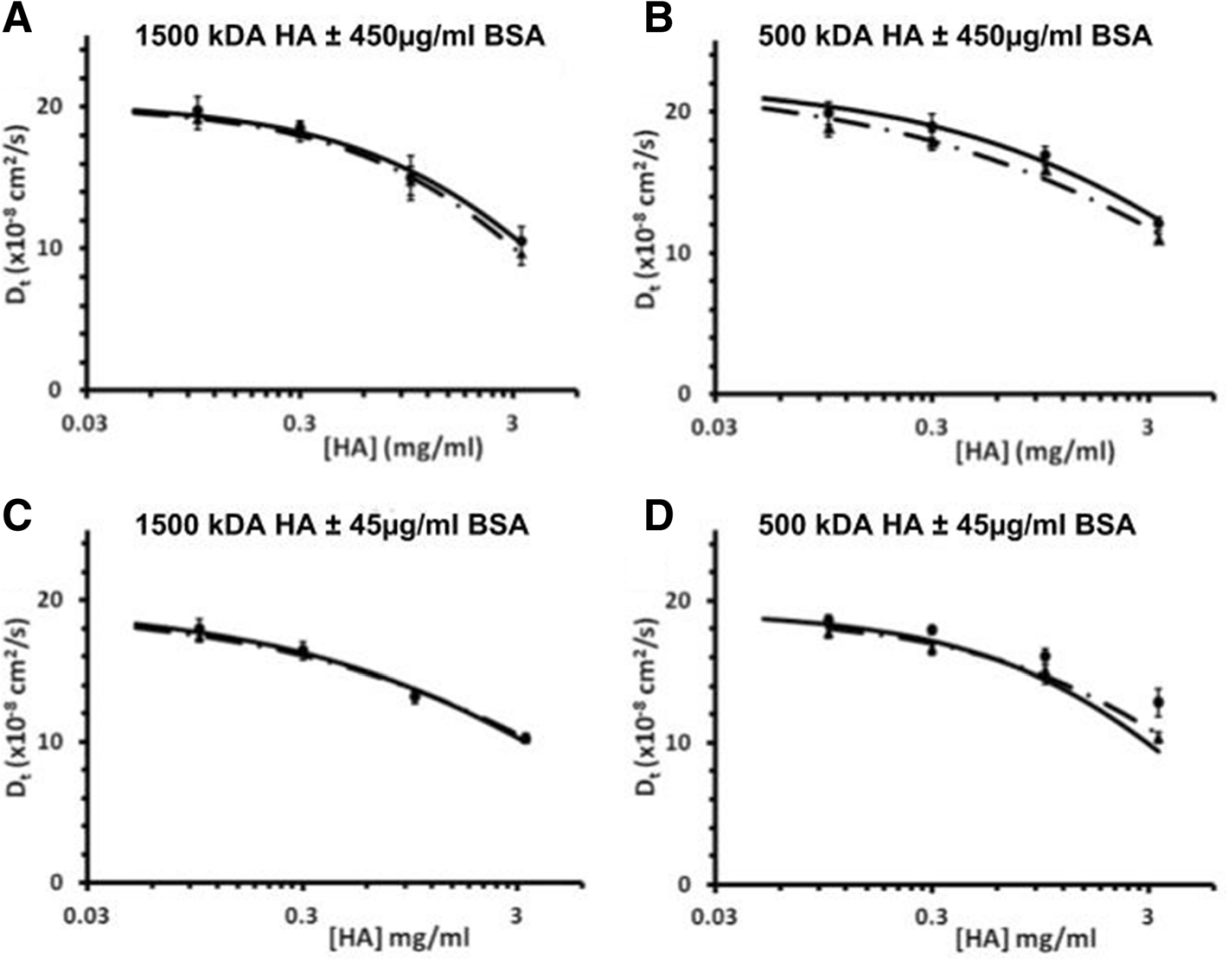
Tracer diffusion coefficients of FITC-dextran (2000 kDa) through 1500 kDa HA solutions ±450 μg/ml BSA (∆ for HA + BSA, ● for HA) (**a**), 500 kDa HA solutions ±450 μg/ml BSA (**b**); 1500 kDa HA solutions ±45 μg/ml BSA (**c**), 500 kDa HA solutions ±45 μg/ml BSA (**d**). Data points are fit to the universal scaling equation (dashed lines = HA + BSA, solid lines = HA). At 450 μg/ml BSA, in both 1500 and 500 kDa HA (**a**, **b**) the diffusion of the tracer was significantly affected by HA concentration (both *p* < 0.01) but not the presence of BSA (*p* = 0.39 and 0.11 respectively), with no interaction effect (*p* = 0.49 and 1.0 respectively). At 45 μg/ml BSA, in both 1500 and 500 kDa HA (**c**, **d**) the diffusion of the tracer through 1500 kDa HA was significantly affected by HA concentration (both *p* < 0.01) but not the presence of BSA (*p* = 0.95 and 0.73 respectively), with no interaction effect (*p* = 0.99 and 0.372, respectively)

**Fig. 5:**
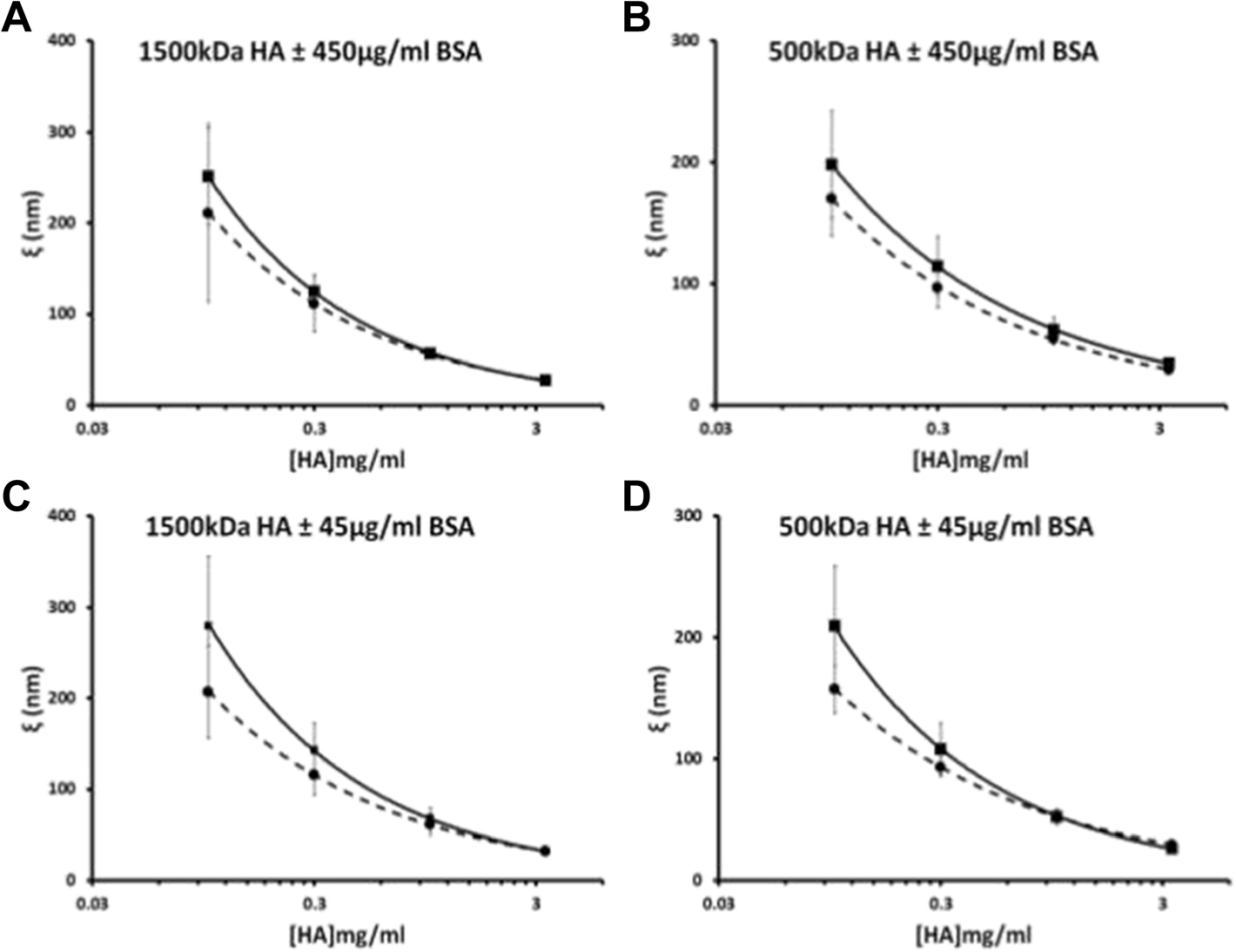
Apparent mesh size distribution (ξ) of 1500 kDa HA ± 450 μg/mL BSA (∆ for HA + BSA, ● for HA) (**a**), 500 kDa HA ± 450 **μ**g/mL BSA (**b**); 1500 kDa HA ± 45 μg/mL BSA (**c**), 500 kDa HA ± 45 **μ**g/mL BSA (**d**). The empirical constants from the scaling equations were used to calculate the average distribution of apparent mesh sizes, ξ, using the correlation length relation (dashed lines = HA + PRG4, solid lines = HA)

BSA did not slow the diffusion of the FITC-dextran tracer through the HA concentration series, either at 450 (Fig. 4a, b) or 45 μg/ml (Fig. 4c, d), and in all cases there was a clear negative exponential decrease in diffusivity for the tracer as HA concentration increases. At 450 μg/ml BSA, in both 1500 and 500 kDa HA the diffusion of the tracer was significantly affected by HA concentration (both *p* < 0.01) but not the presence of BSA (*p* = 0.39 and 0.11 respectively), with no interaction effect (*p* = 0.49 and 1.0 respectively). The measured D_t_^0^ for the FITC-dextran tracer was 20.05 ± 1.36 × 10^−8^*cm*^2^ *s*^−1^, and 22.41 ± 0.83 × 10^−8^*cm*^2^ *s*^−1^, for 1500 and 500 kDa respectively. At 45 μg/ml BSA, in both 1500 and 500 kDa HA the diffusion of the tracer through 1500 kDa HA was significantly affected by HA concentration (both *p* < 0.01) but not the presence of BSA (*p* = 0.95 and 0.73 respectively), with no interaction effect (*p* = 0.99 and 0.372, respectively). The measured D_t_^0^ for the FITC-dextran tracer was 19.58 ± 0.77 × 10^−8^*cm*^2^ *s*^−1^, and 19.25 ± 0.96 × 10^−8^*cm*^2^ *s*^−1^, for 1500 kDa and 500 kDa respectfully. Finally, the addition of BSA to the HA solutions did not appear to change the calculated average apparent mesh size for each experimental set (Fig. 5).

### HA + PRG4 vs. HA + R/a PRG4

Both PRG4 and R/A PRG4 had a similar effect on the HA solution network (data not shown). For both 1500 and 500 kDa HA, the diffusion of the tracer was significantly affected by HA concentration (both *p* < 0.01), but the difference between PRG4 and R/A PRG4 was not significant (*p* = 0.76 and 0.385 respectively), with no interaction effect (*p* = 0.657 and 0.66 respectively). The measured D_t_^0^ for the FITC-dextran tracer for this experimental set was 21.15 ± 1.13 × 10^−8^*cm*^2^ *s*^−1^.

## Discussion

These results demonstrate that PRG4, at physiological concentrations, can significantly alter the solution properties of 1500 and 500 kDa HA; PRG4 significantly decreased the tracer diffusion at all HA concentrations tested here. The physical implication of this finding was characterized by a decreased in the apparent mesh size distribution for each mixture of HA, calculated from the empirical constants (β and ν) from the universal scaling equations successfully fit to the concentration series of HA. This effect was specific to PRG4 and was not observed with BSA, indicating it was not a result of a protein simply being in solution with HA. Interestingly, the reduced permeability observed appeared similar in magnitude for both 450 and 45 μg/ml, and was also not dependent on its tertiary/quaternary structure as the effect remained after R/A of PRG4. As PRG4 and HA are key SF macromolecular constituents that play functional roles in various SF properties (e.g. viscosity, lubrication, solution meshwork), collectively these findings contribute to the understanding of their interaction(s) in solution as well as the function of SF in diarthroidal joints.

The HA and PRG4 used in this study are representative of those in native SF and have been used in other studies. Both the HA MW and concentration range used is relevant to physiological and pathological conditions [10, 22]. The MW of HA used was found to be polydisperse, which may explain the lack of observed difference in tracer diffusion between the 1500 and 500 kDa HA. Due to the unique properties of PRG4, it was difficult to choose the perfect protein control for the tracer diffusion studies. BSA, whose hydrodynamic diameter is ~ 7 nm, was chosen as a practical and relevant protein control that would not interact with HA at the pH employed here, and as it is abundant within SF [23]. Future work could potentially address this limitation by examining other (glyco)protein proteins as potential controls proteins that do not interact with HA. Measurements for D_t_ were shown to have a significant variance between experimental sets, with a standard deviation between measured D_t_ values of 1.4 × 10^−8^*cm*^2^ *s*^−1^. As such, to reduce and control for the effect of the variations between prepared samples, all comparisons of experimental sets (e.g. one replicate of HA vs. HA+ 450 μg/ml PRG4) were performed on the same day from the same prepared HA solutions. Thus, while the day to day variance between experiments can give a sizable variance between identical measurements made on separate days, all analysis of HA vs HA with an additive (PRG4 or BSA) were made between identical sample preparations. The mean calculated diffusion coefficient from all data points (from all experiments) along with the standard error of the mean for the tracer through 1500 kDa HA at 0, 0.1, 0.3, 1.0 and 3.3 mg/ml were 19.29 ± 0.39, 19.57 ± 0.80, 18.27 ± 0.68, 15.12 ± 0.60, 11.51 ± 0.63 × 10^−8^*cm*^2^ *s*^−1^, respectively. Those for 500kD HA at 0, 0.1, 0.3, 1.0 and 3.3 mg/ml were 19.41 ± 0.48, 19.05 ± 0.36, 18.27 ± 0.38, 16.18 ± 0.73, 11.81 ± 0.82 × 10^−8^*cm*^2^ *s*^−1^, respectively. We did not normalize our measurements, so we could accurately show the day to day variance between experiments. Another limitation of the proposed study was the assumption of a strictly 2D diffusing system. This 2D assumption has been shown to be appropriate when using a NA lens, and a large confocal aperture setting [17]. Additionally, any diffusion within the Z plane would be consistent between measurements.

The results from this study agree with those from Gribbon et al. [18], and extend them to include the effect of PRG4. While the results presented here show the same negative exponential trend in tracer diffusion constants in relation to HA concentration, the reported D_t_^0^ of the 2000 kDa FITC-dextran tracer are slightly higher. This could be due variations in data analysis and FRAP parameters (e.g. higher bleaching times, different objective lens), but nevertheless the results from this study are in good agreement with previous research [18, 24, 25]. Furthermore, the predicted trend in tracer diffusion through increasing HA concentration was observed here and the results demonstrate a clear and specific effect of PRG4. Lastly, while future studies could potentially examine direct measurement of mesh size in HA solutions, those calculated here are in good agreement with those previously reported by Gribbon et al. [18]

The mechanism of the PRG4 + HA interaction in solution observed here, and in other studies [2, 5], remains to be completely elucidated. It has been speculated to be one of physical interaction involving non-covalent entanglements, [2] potentially mediated through the hemopexin like and somatomedin B like domains on the C- and N-terminus, [12, 26] respectively, of PRG4. Indeed, hemopexin is able to bind to HA, supporting the plausible mechanism for PRG4 entangling HA molecules through this domain [26]. This highly entangled HA matrix is therefore a conceivable explanation for the observed decrease in tracer diffusion and smaller observed mesh sizes when PRG4 is present. The similar effect observed between native PRG4 and R/A PRG4 was somewhat unexpected since if PRG4 interacts with HA through its globular domains, they would be unfolded by R/A. A potential explanation for this is while PRG4 no longer makes entanglements with HA, it is capable of producing its own solution networks or gels independent (which is well documented in mucins [27]). It would then be these PRG4 networks within the space not occupied by HA, and not entanglements with HA, which cause the denser less permeable networks thus is impeding the diffusion of the FITC-dextran tracers. Indeed, R/A can cause the protein end units of mucins to unfold and expose large hydrophobic domains resulting in aggregation into dense networks [28]. A recent study demonstrating PRG4, but not R/A PRG4, can enhance the viscosity of HA solutions is also consistent with the above interpretation [16]. The hydrodynamic radius of R/A PRG4 has not been reported, however the elution time on a size exclusion column remained similar compared to non-reduced PRG4 suggesting the two have similar hydrodynamic radii [11]. Unfortunately the relative ratio of multimers/monomers in a PRG4 solution has not been quantified, but based on size exclusion chromatographs [7] it seems reasonable to assume they are present in similar (order of magnitude) quantifies. The effect of concentration on this ratio is currently unknown as well. Future studies are required, likely with more than one technique, to further define and validate the model where PRG4 interactions with HA through the hemopexin and somatomedin B domains, entangling HA molecules and creating a tighter and solution matrix with altered rheological properties.

## Conclusions

In conclusion, the implications of this study are important to the function of SF in healthy and diseased joints. Solutions or SF with altered composition of PRG4 and HA can lead to diminished lubrication and viscosity, as well as a larger solution meshwork [15, 16]. As such, an increased understanding of HA and PRG4 interaction and functional synergism would further contribute to the understanding of the role altered SF composition of PRG4 and HA [10] play in OA initiation and progression. Future studies could expand to altering environmental conditions to conform the HA network in predictable ways as well as experimentation to human SF, a more complex HA solution. Given the use of HA as an intraarticular treatment for pain relief in OA, and recent preclinical studies demonstrating the efficacy of full-length recombinant human PRG4 as a treatment for OA [29], these and other findings could contribute to the potential development of new and improved SF engineered supplement as a biotherapeutic treatments for OA.

## Abbreviations

BSA: bovine serum albumin
c: concentration of polymer
d: hydrodynamic diameter of tracer
D_t_: Lateral diffusion coefficient of the tracer
D_t_^0^: Free diffusion coefficient of the tracer
FITC: Fluorescein isothiocyanate
FRAP: Fluorescence recovery after photobleaching
HA: Hyaluronan
J_n_: n order Bessel function
OA: Osteoarthritis
PBS: Phosphate buffered saline
PRG4: Proteoglycan 4
r: radius
R: Total radius of phot bleached area
R/A: Reduction and alkylation
SF: Synovial fluid
t: time
u: scaled intensity of fluorescence
ξ: apparent mesh sizes
